# The global wildland–urban interface

**DOI:** 10.1038/s41586-023-06320-0

**Published:** 2023-07-19

**Authors:** Franz Schug, Avi Bar-Massada, Amanda R. Carlson, Heather Cox, Todd J. Hawbaker, David Helmers, Patrick Hostert, Dominik Kaim, Neda K. Kasraee, Sebastián Martinuzzi, Miranda H. Mockrin, Kira A. Pfoch, Volker C. Radeloff

**Affiliations:** 1 SILVIS Lab, Department of Forest and Wildlife Ecology, University of Wisconsin-Madison, Madison, WI USA; 2 Department of Biology and Environment, University of Haifa at Oranim, Kiryat Tivon, Israel; 3 US Geological Survey, Geosciences and Environmental Change Science Center, Lakewood, CO USA; 4 Geography Department, Humboldt-Universität zu Berlin, Berlin, Germany; 5 Integrative Research Institute on Transformations of Human-Environment Systems, Humboldt-Universität zu Berlin, Berlin, Germany; 6 Institute of Geography and Spatial Management, Faculty of Geography and Geology, Jagiellonian University, Krakow, Poland; 7 Northern Research Station, US Department of Agriculture Forest Service, Baltimore, MD USA

**Keywords:** Environmental impact, Ecosystem ecology, Sustainability, Natural hazards, Fire ecology

## Abstract

The wildland–urban interface (WUI) is where buildings and wildland vegetation meet or intermingle^1,2^. It is where human–environmental conflicts and risks can be concentrated, including the loss of houses and lives to wildfire, habitat loss and fragmentation and the spread of zoonotic diseases^3^. However, a global analysis of the WUI has been lacking. Here, we present a global map of the 2020 WUI at 10 m resolution using a globally consistent and validated approach based on remote sensing-derived datasets of building area^4^ and wildland vegetation^5^. We show that the WUI is a global phenomenon, identify many previously undocumented WUI hotspots and highlight the wide range of population density, land cover types and biomass levels in different parts of the global WUI. The WUI covers only 4.7% of the land surface but is home to nearly half its population (3.5 billion). The WUI is especially widespread in Europe (15% of the land area) and the temperate broadleaf and mixed forests biome (18%). Of all people living near 2003–2020 wildfires (0.4 billion), two thirds have their home in the WUI, most of them in Africa (150 million). Given that wildfire activity is predicted to increase because of climate change in many regions^6^, there is a need to understand housing growth and vegetation patterns as drivers of WUI change.

## Abstract (web summary)

A global assessment shows that the wildland–urban interface occurs on all continents, showing its broad-scale patterns and providing a basis for future research on dynamics and socioeconomic and biophysical processes.

## Main

Humans have greatly affected the Earth’s land surface in recent centuries^7–11^. In particular, the expansion of the built environment and the growth of settlements and their long-term resource requirements have been dramatic across the globe^12–14^. The growth of settlements can have remote effects via teleconnected processes^15,16^ but the most immediate human–environmental conflicts arise where buildings are built in or near wildland vegetation, an area known as the wildland–urban interface (WUI)^1,17^. The WUI is widespread across Australia, Europe and North America^18–22^ and there is evidence for WUI in some other countries^23–26^. However, the worldwide distribution of the WUI is unknown^2^.

The WUI is a desirable place to live for many people as a result of its proximity to natural amenities but it is also an area of manifold hazards to both humans and natural ecosystems. Wildfires are a particular threat to houses and lives, often caused by human ignition and facilitated by altered fire regimes where settlements sprawl into fire-dependent ecosystems. The availability of buildings themselves as fuel, along with swiftly moving fire, makes evacuations difficult^19,27–29^. Indeed, the number of wildfires has increased in the WUI over the past few decades^2^ owing to both housing growth and climate change. Other hazards to humans or their environment include the loss of biodiversity and carbon storage due to habitat loss and fragmentation, predation of wildlife by cats and dogs, light and noise pollution, the introduction of invasive species, an increased risk for the spread of zoonotic diseases and changes in hydrology^3,30–33^. Quantifying any of these hazards requires a consistent global assessment of the WUI. This is particularly important because the number of exposed buildings and people in the WUI is expected to grow as population grows and because climate change is expected to further increase the risk for many of these hazards, such as higher wildfire frequency and intensity^18,34,35^.

Here, we present a global map of the 2020 WUI at 10 m resolution using a globally consistent and validated approach based on remote sensing-derived datasets of building area^4^ and wildland vegetation^5^. We distinguished between two types of WUI: intermix WUI (where buildings and wildland vegetation intermingle) and interface WUI (where buildings are close to large wildland vegetation patches). We further distinguished between WUI dominated by forest, shrubland and wetland versus that dominated by grassland. We then summarized population and biomass in the WUI for each country and biome, using the biome definition of ref. ^36^. To identify areas of increased fire hazard in the WUI, we assessed wildfire occurrence using two remote sensing-based datasets—Moderate Resolution Imaging Spectroradiometer (MODIS) Active Fire data for 2003–2020 and Visible Infrared Imaging Radiometer Suite (VIIRS) Active Fire data for 2013–2020. Our identification of WUI types by dominant land cover allowed for a regionalized evaluation of fire hazard in the WUI. For example, whereas natural grasslands exhibit frequent wildfires in some of the world’s WUI, wildfires are not a concern where grasslands are highly managed pastures. In contrast, both managed and wild forests provide fuel for wildfires.

We found that the total global WUI area in 2020 was 6.3 million km^2^ or 4.7% of global land area, which is an order of magnitude larger than the global urban area^37^ or twice the size of India. The global land share of intermix and interface WUI is 3.6% (4.8 million km^2^) and 1.1% (1.5 million km^2^), respectively. Two thirds of the overall WUI area are dominated by forests, shrublands and wetlands, versus one third by grasslands. Globally, 3.5 billion people live within the WUI (1.7 billion in intermix and 1.8 billion in interface WUI) and two thirds of those live in WUI dominated by forests, shrublands and wetlands. In total, nearly half of all buildings and people on the globe are potentially affected by the human–environmental hazards that are concentrated in the WUI. However, only 4.1% of the total aboveground living plant biomass occurs within the WUI, with most of it in the intermix WUI.

**Fig. 1:**
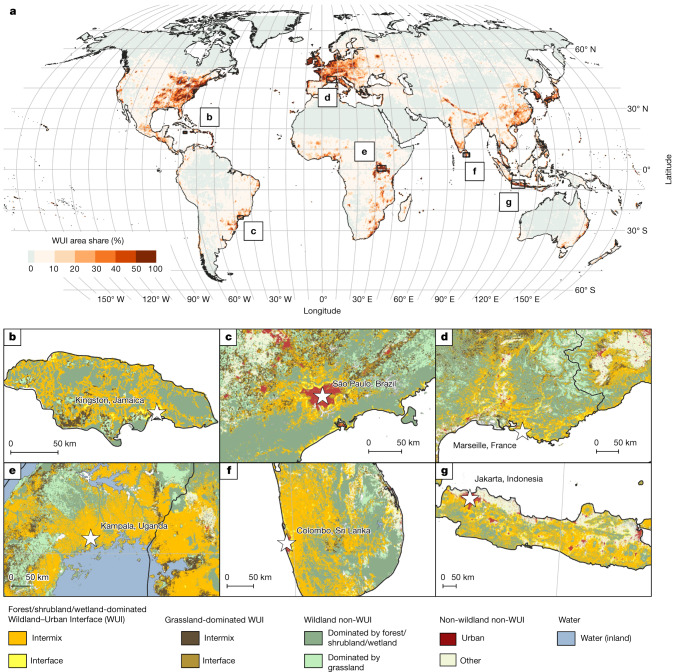
The global wildland–urban interface. **a**, Area share (%) of the WUI in about 2020 per hexagon with 50 km diagonal length. **b**–**g**, WUI and non-WUI map at 10 m resolution for parts of Jamaica (**b**), Brazil (**c**), France (**d**), Uganda (**e**), Sri Lanka (**f**) and Indonesia (**g**). Interactive global map at https://silvis.forest.wisc.edu/data/globalwui. Land surface/country masks from https://www.naturalearthdata.com.

The WUI occurs on all continents. However, within continents, the distribution of the WUI is highly uneven. Large WUI areas occur along the Pacific coast of North America; in eastern North America and the Caribbean; along the Brazilian coast; across Europe; in West, South and East Africa, including Nigeria and Uganda; in Southeast Asia, including India, China, Indonesia and Japan; and in Australia (Fig. 1a). In some of these places, such as in California, Mediterranean Europe or South Africa, the WUI has been well studied because many buildings and people are affected by wildfires there^38,39^. In many other places, however, including East Africa, Brazil or Southeast Asia, widespread WUI has not been reported. Among the two most populated countries in the world, China has large WUI areas in southern and eastern regions, which are previously undocumented in the literature. However, India has much smaller WUI area in the southeast and the Himalayas, probably because cropland density is high in other regions^40^, not providing enough wildland vegetation to create WUI. The area-adjusted overall accuracy of our WUI map is between 79.6% (when distinguishing all WUI classes) and 82.0% (when distinguishing WUI from non-WUI; Supplementary Data A).

**Fig. 2:**
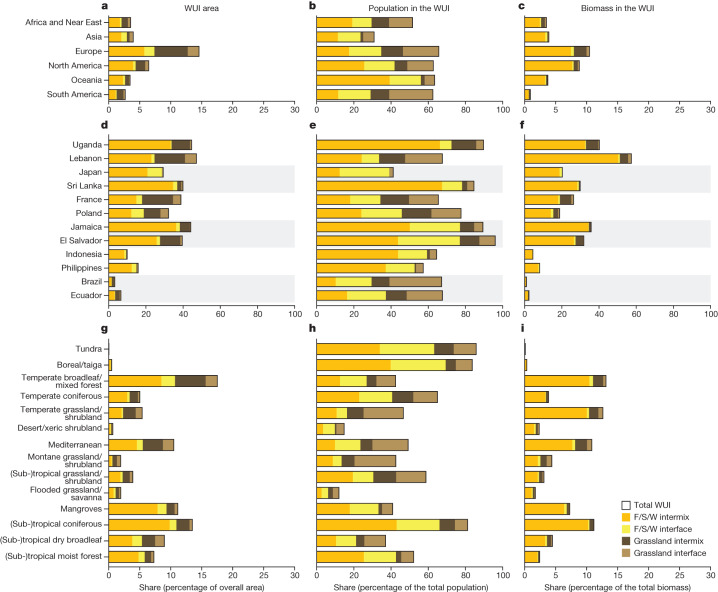
WUI area, population and biomass by world region, selected countries and biomes. **a**–**i**, Share of the WUI area in the total land area (**a**,**d**,**g**), share of population within the WUI (**b**,**e**,**h**) and share of biomass within the WUI per world region (**a**–**c**), selected country (**d**–**f**) and biome (**g**–**i**) (by latitude) as defined by ref. ^36^. Shaded grey bars in **d**, **e** and **f** highlight world regions. F/S/W: dominated by forest/shrubland/wetland. Land surface/country masks from https://www.naturalearthdata.com. Source Data

The characteristics of the WUI vary considerably among continents. The WUI covers only 3% of South America but 15% of Europe. Europe and Asia have especially high shares of interface WUI area, whereas intermix WUI dominates in North America. South America is the only world region where grassland WUI area dominates, whereas Asia has the least. In South America, 33% of the population live in grassland-dominated WUI but only 7% in Asia. In Oceania, 56% of the total population lives in WUI dominated by forest, shrublands and wetlands, compared to only 24% in Asia. Europe has the largest share of its biomass, 10.5%, within the WUI (Fig. 2a–c). Seventy per cent of the global WUI area is in very low or low density rural areas and only 8% in urban clusters and centres (Extended Data Table 1, classes according to ref. ^41^). However, this pattern differs strongly by world region: in North America, 84% of the WUI is rural (5% in urban areas) but in Asia only 53% (14% in urban areas). Lastly, the WUI occurs in countries across all income classes (Extended Data Table 2).

**Extended Data Fig. 1:**
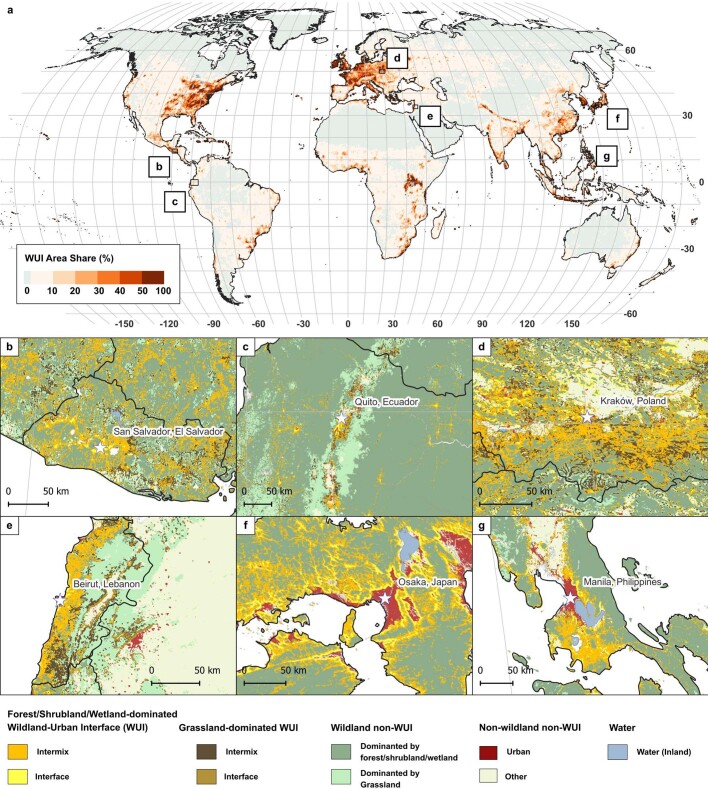
The global Wildland–Urban Interface. **a**, Area share (%) of the Wildland–Urban Interface (WUI) in ca. 2020 per hexagon with 50 km diagonal length. **b**–**g**, WUI and non-WUI map at 10 m resolution for parts of El Salvador (**b**), Ecuador (**c**), Poland (**d**), Lebanon (**e**), Japan (**f**), and the Philippines (**g**). Interactive global map at https://silvis.forest.wisc.edu/data/globalwui/.

We selected 12 hotspot countries on the basis of WUI area share and wildfire occurrence for closer examination: Uganda, Lebanon, Sri Lanka, Japan, France, Poland, Jamaica, El Salvador, Indonesia, the Philippines, Brazil and Ecuador (Fig. 1b–g, Extended Data Fig. 1 and Fig. 2d–f). Uganda, Sri Lanka, Jamaica and El Salvador have an exceptionally high share of population in the WUI (>80%) and many people there were affected by fires since 2003 (for example, 8.7 million in Uganda and 1.4 million in El Salvador). Lebanon has an exceptionally high share of biomass in the WUI (60%) because most WUI occurs near its coastal regions where biomass is concentrated. In Japan, more than half of all wildfires occurred within the WUI and most of the people living in the WUI live in the interface because settlements are generally well demarcated and abut wildland vegetation. France and Poland have an especially high share of WUI area and population in the grassland WUI. In Indonesia, the Philippines, Brazil and Ecuador, WUI area share is small but high proportions of people live in those small WUI areas and are affected by wildfires (0.7 to 13.1 million, depending on the country). Those different WUI patterns reflect the diversity of reasons for both WUI development and wildfire occurrence and highlight that different management responses are required to mitigate the human–environmental conflicts that are concentrated in the WUI^3^.

**Extended Data Fig. 2:**
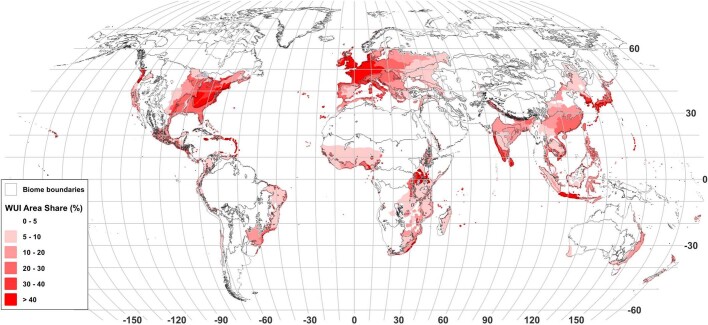
WUI area share by biome. Per cent area share of the total of the four mapped WUI classes per biome. Black lines: Biome boundaries as defined by Olson et al. (2001)^36^. Map projection: Robinson. Grid coordinates: WGS 84.

Among biomes, the WUI is highly concentrated in a few (Fig. 2g–i and Extended Data Fig. 2). The temperate broadleaf and mixed forest biome covers only 9% of global land area, yet contains 35% of total WUI area. Similarly, subtropical and tropical moist broadleaf forests represent only 15% of the global land but contain 26% of WUI area. In contrast, deserts and xeric shrublands cover 20% of the land area but contain only 3% of the global WUI area. We also observed large differences in population patterns: in both boreal forests/taiga and in subtropical and tropical coniferous forests over 80% of the population lives in the WUI, whereas in the deserts and xeric shrublands, only 10% does. The distribution of WUI area across biomes is important because WUI-related hazards, such as wildfires and their effects on people probably differ among biomes. Wildfire hazard is higher either where the WUI is widespread and where many people are affected by wildfire (such as in subtropical and tropical moist broadleaf forests) or where WUI area itself is small but both people and wildfires are concentrated there (such as boreal forests/taiga and mangroves that are highly vulnerable to fire). Wildfire hazard is also driven by the available biomass. In both the broadleaf/mixed forests and in subtropical and tropical coniferous forests, a substantial share of their biomass is in the WUI (about 13% in both biomes) as a result of their high overall share of land in the WUI (18% and 14%, respectively). By contrast, in the temperate grassland, savanna and shrubland biome, the overall WUI area share is low but a large portion of the biomass occurs in vegetation-rich coastal areas, which is also where the WUI is concentrated.

**Fig. 3:**
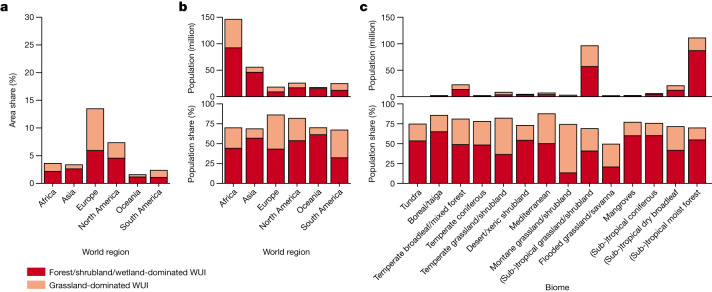
Wildfire in the WUI. **a**, Share of wildfire area within the WUI. **b**, Share of population (%) and number of all people that experienced wildfire within 1 km of their home that are within the WUI by world region. **c**, Share of population (%) and number of all people affected by wildfire that are within the WUI by biome (by latitude) as defined by ref. ^36^. Numbers according to MODIS active fire detection from 2003 to 2020. We analysed active fire point data in a 1 km grid and we considered all grid cells with at least one fire as being wildfire areas and all people living in a grid cell where a fire occurred 2003–2020 as affected by wildfire. Relative patterns are confirmed using VIIRS Active Fire data from 2013 to 2020 (Supplementary Data B–E). Source Data

Wildfires are of increasing concern across the globe, as their frequency, intensity and season-length have increased because of climate change, more human ignitions and rising fuel loads^42^. Wildfires are particularly problematic in the WUI and cause substantial losses of houses and lives there^43^. Indeed, more than two thirds of all people affected by wildfires during 2003–2020 (those experiencing a fire within 1 km of their homes) live in the WUI. This is partly because population density in the WUI is higher than in non-urban non-WUI areas but nevertheless substantial because only a small share of all global wildfire occurrences was directly in the WUI (Fig. 3a). Effects of WUI wildfires on the population differ among world regions and countries. In North America, 85% of the population affected by wildfire lives in the WUI but in Africa only 55% (nearly 150 million) does. In all world regions, except Europe and South America, most people were affected by wildfires that occurred in WUI dominated by forests, shrublands and wetlands (Fig. 3b). This suggests that, despite the WUI’s small overall area, and, despite the comparatively rare occurrence of wildfires, buildings and people in the WUI may face an elevated wildfire hazard across the world.

The role of wildfires in the WUI differs among biomes but wildfire distribution suggests increasing effects of wildfires on people in the future. In some biomes, for example, in the tundra or in deserts and xeric shrublands, wildfires in the WUI are not a widespread phenomenon and relatively few people are affected (Fig. 3c). Some biomes, for example, Mediterranean forests, woodlands and shrublands, are small in area but are hotspots of recurring severe wildfire and destruction^42^. In other biomes, the large share of population living in the WUI (for example, 80% in boreal forests/taiga) and the fact that most people affected by wildfire during 2003–2020 lived in the WUI suggests that changing wildfire regimes could quickly increase the likelihood of wildfire exposure in the future. Particularly, there is a high probability that temperate broadleaf and mixed forests and subtropical and tropical moist broadleaf forests, the biomes with the largest WUI area and home to 130 million people previously affected by wildfire in the WUI, will experience increased fire hazard towards the middle of the twenty-first century if rising trends in wildfire fire frequency and intensity continue^6,44–46^.

## Discussion

The WUI is where people live within or near wildland vegetation. We found that the WUI covers nearly 5% of the global land area, even though the WUI has not been a class in any previous global land cover or land-use maps. We quantified the global extent of the WUI at high spatial resolution, characterized it by dominant land cover and related it to wildfires of the last two decades. Our analysis yielded three principal insights. First, we found that the WUI is a global phenomenon. Although previous work showed that the WUI is widespread in Mediterranean Europe, the United States and Australia^2^, our results show large WUI areas in all continents, including previously undocumented hotspots in eastern Asia, East Africa and parts of South America. Second, the WUI is highly diverse in terms of population density, biomass quantity and dominant vegetation type. Third, the WUI is where wildfires affect the most people. Globally, two thirds of all the people that experienced wildfires live in the WUI. Among our WUI hotspots with frequent wildfires, many lack assessments of wildfire regimes, settlement patterns and wildfire risk, as is true for many WUI hotspots where wildfires are likely to become more prevalent as a result of changing climatic conditions.

Local and regional patterns of the WUI are highly variable. We found major differences in the proportion of area that is WUI in different countries, how many people live there, how much biomass occurs in the WUI and what the dominant land cover is. Although some WUI areas are well-known due to a history of disastrous wildfires (for example, Mediterranean areas of California and Europe and in Southeastern Australia), we found many more places where the WUI is widespread. Some of these also have frequent wildfires, whereas other hazards and conflicts may dominate in other WUI areas. Furthermore, the patterns of the WUI vary greatly, from large continuous WUI areas in East Africa, stretching over hundreds of kilometres, to small and patchy WUI in the heterogeneous landscapes of Mediterranean Europe (Fig. 1d,e). Irrespective of whether a WUI area is large or small, it is likely to be a current or future hotspot of human–environmental conflicts affecting many people and many different types of ecosystems. As fire-prone areas expand globally, our WUI data can help guide proactive actions to prepare for future wildfire in the WUI and tailor such preparations according to the dominant vegetation types and associated fire regimes.

Wildfire damage to buildings is a global problem and we show that most people who experienced wildfire live in the WUI. This applies even in areas where wildfires are common but population density is low, such as in boreal forests or where wildfires are rare and few people live, such as in deserts. However, the subtropical and tropical moist forests, subtropical and tropical grasslands and shrublands and temperate broadleaf and mixed forests are the biomes where the most people live in the WUI and experienced wildfires. In these biomes, a future increase in the exposure of people to wildfire is probably due to (1) population and WUI growth^45^; and (2) an increasing frequency of extreme weather events due to climate change, such as longer and more severe drought, causing lower fuel moisture and a higher frequency and severity of wildfires^46^. How climate change affects wildfires will differ by vegetation type though. Grasslands, for example, can respond rapidly even to incremental climate change^47^ and herbaceous fuels can increase rapidly after periods of high precipitation. Given that 15% of the global population lives in the grassland-dominated WUI, more intensive grassfires could become a big challenge for both wildfire preparedness and response^44^.

Our WUI maps provide an accurate and high-resolution global perspective on a land-use type that is home to nearly half of the global population. The overall accuracy of our WUI maps was consistently high across regions and classes with only slight differences caused by uncertainties in the underlying building and land cover datasets (for example, 79% for Oceania versus 86% in Africa). Our WUI maps enable researchers to identify WUI hotspots where climate change, population growth, land-use change and increasing wildfire and other hazards are likely to cause the most pressing problems. Our maps are valuable because they offer a consistent global assessment at a resolution that is sufficiently fine to inform local and regional management, in addition to showing how global fire regimes are caused by and affecting humans^48^. Future research will need to assess wildfire risk in the WUI in detail because that risk and the associated social vulnerability are affected by a multitude of factors, including land management practices, ecological and economic value, community preparedness, natural disturbance regimes, regional precipitation, temperature and vegetation patterns and wildfire management and prevention^49^. For example, our maps treat both wild steppes and managed pastures as grasslands, yet the former are highly susceptible to wildfire whereas the latter are not. Similarly, our maps do not distinguish between natural forests and plantations, yet forest type can affect fire dynamics and wildfire likelihood. In WUI areas, fire risk can either increase as a result of higher fuel loads and more human ignitions or decrease as a result of fire suppression and fuel treatments, especially when buildings and people are threatened^45^. The global WUI is, and will be, an area of both human–wildlife conflicts and coexistence. It is, thus, a key area to discover how to shape resilient, sustainable and livable settlements, in addition to minimizing human–environmental conflicts^50^. Although fine-scale research is required to understand local drivers of WUI patterns, our globally consistent assessment highlights that WUI occurs on all continents, reveals its broad-scale patterns and provides a basis for future research on global WUI dynamics and the socioeconomic and biophysical processes that make the WUI unique.

## Methods

### Defining the wildland–urban interface

Although the WUI is defined in different ways for different regions and applications^19–24,51–54^, we used the conceptual WUI definition of the US Federal Register^53^, first operationalized by ref. ^1^, which is the most widely used WUI definition in the United States and many other countries^1,19–21,54^. This approach defines the intermix WUI as areas with more than 6.17 buildings per km^2^ (or one building per 40 acres) and a wildland vegetation area share of greater than or equal to 50%. The interface WUI is defined as areas with more than 6.17 buildings per km^2^ but less than 50% wildland vegetation that lie in proximity (less than 2.4 km) of a large patch (at least 5 km^2^) of wildland vegetation (with a share of more than 75%). The minimum patch size excludes small urban parks from wildland vegetation^1^. The minimum distance of 2.4 km (1.5 miles) is included in the US Federal Register definition^53^ and represents the distance embers can fly during a wildfire^55^.

**Extended Data Fig. 3:**
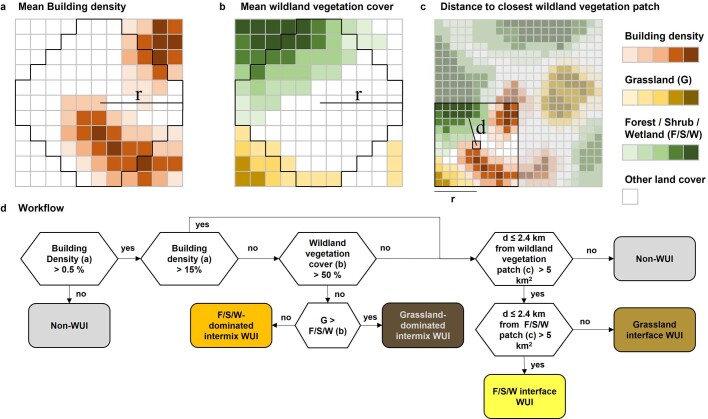
The Wildland–Urban Interface mapping workflow. Classifying the Wildland–Urban Interface based on **a**, moving window mean building density and **b**, wildland vegetation in a circular kernel (r = radius = 500 m, black outline) and **c**, distance to the closest large wildland vegetation patch (d = distance = 2,400 m). G = grassland, F/S/W = forest/shrubland/wetland. **d**, Combining building density and wildland vegetation to map the Wildland–Urban Interface.

We further extended this WUI classification approach by stratifying WUI areas on the basis of the dominant land cover, into intermix or interface WUI dominated by forest, shrubland and wetland versus WUI dominated by grassland (Extended Data Fig. 3). We added that stratification because grasslands are among the most diverse and dynamic land cover types across the globe, with a large range of management practices, from wild steppe to managed pasture, almost resembling agricultural use^56,57^. As a result, grasslands in a given place may or may not cause wildfire risk in the WUI, which is why previous national-level WUI maps purposefully either included^58^ or excluded^21^ grasslands. Our separation of grassland-dominated WUI supports subsequent map interpretation.

### Wildland vegetation and building data

We used two freely available global high-resolution datasets on land cover and buildings to map the WUI. Both datasets are derived from Earth observation satellite images and come in a raster format.

We used the European Space Agency WorldCover dataset to capture land cover information^5^. It is representative for 2020 (v.100) and provides land surface cover information distinguishing 11 classes globally with 10 m resolution. The overall accuracy of this dataset is about 75% (ref. ^59^). The information was derived from Sentinel-1 and Sentinel-2 satellite imagery using an ensemble of gradient-boosting decision trees with expert rule-based postprocessing to map many land cover classes at the same time (Supplementary Information)^5^.

We used the Global Human Settlement GHS-BUILT-S—R2022A dataset (hereafter, GHS-BUILT-S) as a reference for building location and density^4^. GHS-BUILT-S is representative for 2018 and provides pixel-wise estimates of built-up surface area (from 0% to 100% in steps of 1%) globally at 10 m resolution. The dataset contains all building types (with residential, commercial, industrial, agricultural, service or other purposes). The information was derived from Sentinel-2 satellite imagery using a symbolic machine learning approach designed to accurately capture built-up surface area (Supplementary Information).

**Extended Data Fig. 4:**
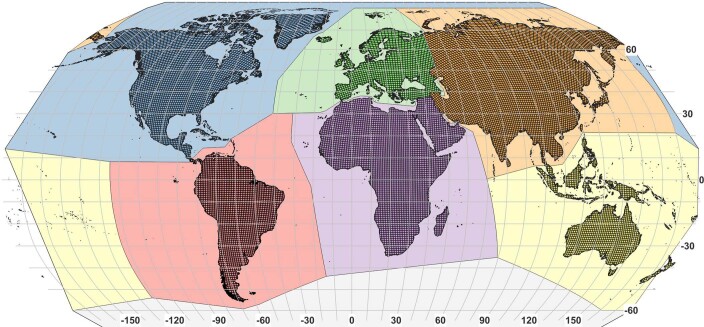
World regions and tiling scheme, EQUI7 reference grid. Tile counts: Africa – 3814 (purple), Asia – 4547 (orange), Europe – 1374 (green), North America – 3155 (blue), Oceania – 2026 (yellow), South America – 2055 (red). Adapted from Bauer-Marschallinger et al. (2014)^62^. Map projection: Robinson. Grid coordinates: WGS 84.

We organized all spatial data in a data cube structure^60,61^ using the FORCE software^61^, matching the first tier of the EQUI7 reference grid^62^. This grid defines an equidistant projection for seven world regions (Africa, Antarctica, Asia, Europe, North America, Oceania and South America) divided into 100 km tiles. The grid facilitates mass data storage and efficient processing, meanwhile avoiding spatial grid oversampling and raster distortion. We used tiles over land for all EQUI7 world regions excluding Antarctica (Extended Data Fig. 4 and Supplementary Information).

### Mapping the wildland–urban interface

We implemented a globally consistent workflow to map the WUI. Building on WUI-mapping approaches that use census block^19^ or building location data^54^, we made some adaptations for our raster data approach. Most notably, we could not apply the building density threshold of 6.17 km^−2^.

We reclassified the land cover data into wildland versus non-wildland. Wildland vegetation included tree cover/forest, shrubland, grassland, herbaceous wetland, mangroves and moss and lichen. Non-wildland vegetation included cropland, built-up area, bare soil and sparse vegetation, snow and ice and water. For the interface WUI, we performed two reclassifications, where grassland was either included as wildland vegetation or not. Accordingly, we mapped two sets of large vegetation patches. We used the reclassified datasets to compute the wildland vegetation share within a circular kernel for intermix mapping (radius of 500 m following precedent)^54^. We also identified patches of more than 5 km^2^ where wildland vegetation share is greater than 75% for interface mapping. Pixels within 2,400 m (following precedent^54^) of large vegetation patches were included as interface WUI.

On the basis of initial tests, we set building density to zero where slope is more than 25° (based on a gap-filled SRTM^63^/ASTER^64^ digital elevation model) or where intra-annual water occurrence in the Global Water Surface dataset^65^ is more than 20%, that is, where water was present during at least 20% of the year to reduce commission errors on steep bare rock and in temporary river beds where false detections of buildings were more common (Supplementary Information). Also, we only considered pixels with an estimated building density of more than 20%, thereby removing areas with very low building density for which data accuracy can be limited. We defined all pixels with an aggregated building density greater than 0.5% in their surrounding (500 m radius) as candidate WUI pixels. Compared to the commonly used definition of 6.17 buildings per km^2^, this threshold is usually slightly higher (depending on local building sizes). We chose this threshold to avoid WUI commission errors in low building density areas, which means that our WUI estimates are conservative. Similarly, we defined pixels with an average building density greater than 15% in a 500 m radius as having an urban character. These areas, for example, densely vegetated and high-density suburban environments, could not be classified as intermix WUI because urban vegetation often differs from wildland vegetation in terms of species identity, management practices and habitat restrictions and stronger fire control systems are in place that prevent fires. WUI mapping was subsequently performed as illustrated in Extended Data Fig. 3.

The WUI maps were masked where land cover was water. For intermix WUI, we determined the dominant land cover type within a pixel based on the area share of wildland vegetation. We distinguished pixels dominated by forests, shrubland and wetlands from those dominated by grassland.

We identified candidate hotspot countries as the top ten countries in their respective world region with the highest WUI area share, that had more than 20% of their wildfire area within the WUI and were more than 10,000 km^2^ in size. Among these, we selected the two countries with the most people affected by wildfire in the WUI. If their borders were within 200 km, we replaced the second-ranked country with the third-ranked (until border distance greater than 200 km).

### Population, biomass and fire

We analysed the extent and distribution of the global WUI and also calculated the population living in the WUI, proportion of biomass in the WUI and WUI area affected by wildfire.

For population data, we analysed the Global Human Settlement Population dataset (GHS-POP^66^) that represents population per grid cell, with 100 m resolution. This dataset is based on the building density dataset we used to map the WUI but excludes non-residential buildings. It was created by disaggregating census data to grid cells using building density as weight. We computed area-weighted summaries of population data.

For biomass, we analysed global maps of aboveground biomass carbon density for 2010 (ref. ^67^), with 300 m resolution. We converted biomass carbon density (MgC ha^−1^) to mass (kg) and applied a factor of two to convert carbon equivalent mass to dry matter biomass^68^. We computed area-weighted summaries of biomass data.

For wildfire data, we analysed the MODIS Collection 6.1 Active Fire dataset (MCD14ML)^69^ and extracted grid cell-based fire frequency data from 2003 to 2020 (the years with complete data records for both Aqua and Terra). We selected only fires categorized as vegetation fires and excluded those representing active volcanoes and static land sources such as gas flares. Furthermore, we distinguished wildfires from agricultural or structural fires by only including fires for which the share of wildland vegetation in that MODIS pixel was more than 50% according to the WorldCover dataset. We reduced fire frequency data to fire presence by setting many fire occurrences within one grid cell to one. We defined a grid cell with fire presence as an area affected by wildfire. We used the MODIS Active Fire dataset because it provides the longest consistent spatially explicit global time series information of fire. However, MODIS active fire data have some limitations, for example caused by the wide sensor swath of 2,230 km, which can result in pixel area differences between nadir and the swath edges of a factor of 10, thereby potentially underestimating fire area at the swath edges^70^. This is a particular issue as active fires are detected by thermal anomalies that, if classified as fire, are represented by a single point in the centre location of the pixel. Furthermore, its nominal pixel resolution of 1,000 m can result in the non-detection of small fires, particularly in low tree cover areas. This is why we also analysed data from the VIIRS Active Fire product which has 375 m spatial resolution since 2013. VIIRS data are comparable to the MODIS product but overcome some of its challenges as a result of higher resolution and narrower sensor swath^71^. We compared fire area and population affected by fire derived from VIIRS (2013 to 2020) or the MODIS data (2013–2020 for comparison and 2003–2020 for our main summary statistics).

### Area correction

Where applicable, we used a pixel-based area-correction factor when computing area statistics to adjust skewed area statistics caused by our projection system (Supplementary Information).

### Accuracy assessment and uncertainty

We evaluated the accuracy of the global WUI maps thoroughly using a stratified random sample^72^. Validation sites were stratified on the basis of the mapped area shares of our five classes: forest/shrub/wetland-dominated and grassland-dominated intermix and interface WUI and non-WUI. We conducted our validation independently for each of the six world regions based on expert-opinion reference data derived from the visual interpretation of submetre to metre resolution satellite imagery available in Google Earth.

According to ref. ^72^, the number of required validation sites is based on the mapped area proportion *W*_*i*_ of each validated class *i*, the target user’s accuracy *U*_*i*_ and the target standard error *S* for the estimated overall accuracy (equation (1)).

1 $$n={\left(\frac{\mathop{\sum }\limits_{i=0}^{5}\left({W}_{i}\times \sqrt{{U}_{i}\times \left(1-{U}_{i}\right)}\right)}{S}\right)}^{2}$$

**Extended Data Fig. 5:**
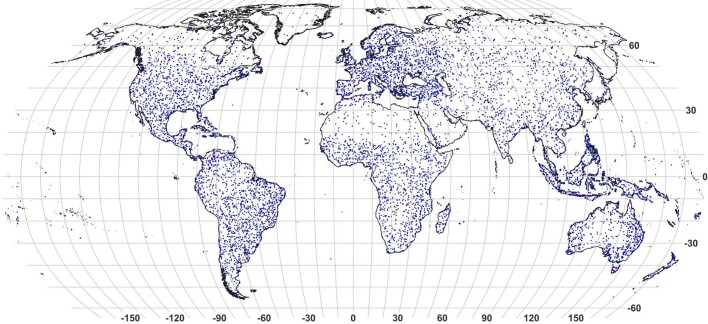
Global Wildland–Urban Interface, Validation Sites. Global distribution of all validation sites, 1,504 per world region for a total of 9,024, world regions defined according to Extended Data Fig. 4. Map projection: Robinson. Grid coordinates: WGS 84.

The number of sites *n* was drawn for each world region. The sites were then equally allocated to the five classes. A class-area proportion of the distribution would have complicated data handling, as non-WUI was expected to be by far the dominant class. The total number of validation sites was 1,504 per world region, that is, 300 or 301 per world region and class, based on a target user’s accuracy of 0.75 and a target standard error of 0.01. The sites were randomly drawn within the respective strata (Extended Data Fig. 5).

**Extended Data Fig. 6:**
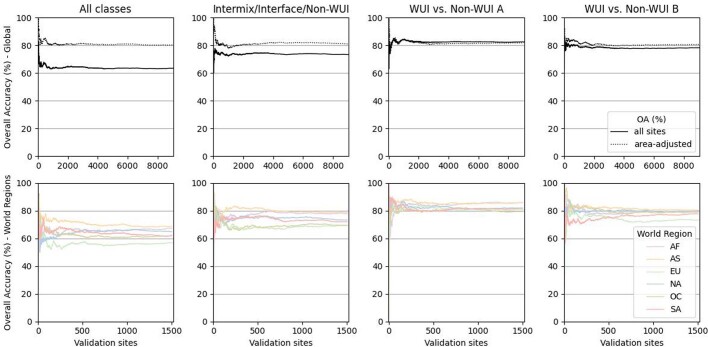
Overall Accuracy of global Wildland–Urban Interface (WUI) mapping. Iterative overall and area-adjusted accuracy (%) globally (top) and by world region (bottom). Columns represent different class aggregations. All classes (left): all mapped classes individually (non-WUI, forest/shrubland/wetland-dominated intermix WUI, grassland-dominated intermix WUI, forest/shrubland/wetland-dominated interface WUI, grassland -dominated interface WUI). Intermix/Interface/Non-WUI (centre left): intermix and interface classes aggregated respectively. WUI vs. Non-WUI A (centre right): all WUI classes aggregated. WUI vs. Non-WUI B (right): aggregates forest/shrubland/wetland-dominated WUI as WUI, and grassland-dominated WUI as non-WUI.

The overall area-adjusted mapping accuracy when distinguishing WUI versus non-WUI classes was 82.1% (Extended Data Fig. 6). The area-adjusted overall accuracy when all five classes were separately assessed was 79.6%. Class-wise user’s and producer’s accuracies ranged considerably and so did overall accuracies without area-adjustment (Supplementary Data A). Area-adjusted accuracy is largely affected by the high area share and high user’s accuracy of the non-WUI class, whereas all WUI classes have a minor area share across the globe. The overall accuracy varied only slightly among the world regions (between five and ten percentage points), with no clear recurring patterns. The overall accuracy between different interpreters differed by similar margins. Only 3% of all validation points were labelled as ‘uncertain’ during the validation process. The quality of the WUI map is largely a function of the quality of the underlying land cover data. For example, despite the overall high accuracy of the ESA WorldCover product, the user’s accuracy of shrublands, one of the key land cover types for WUI mapping, is only 39% (ref. ^59^). The quality of our WUI map also depends on the quality of building data. However, we found that because WUI requires only to be above the minimum building density threshold, even fairly widespread omission errors in areas with scattered buildings typically do not lead to missed WUI. On the other hand, in areas where small, isolated buildings are missed, the mapped WUI area was not greatly affected either because such isolated buildings do not form WUI even when mapped correctly.

**Extended Data Fig. 7:**
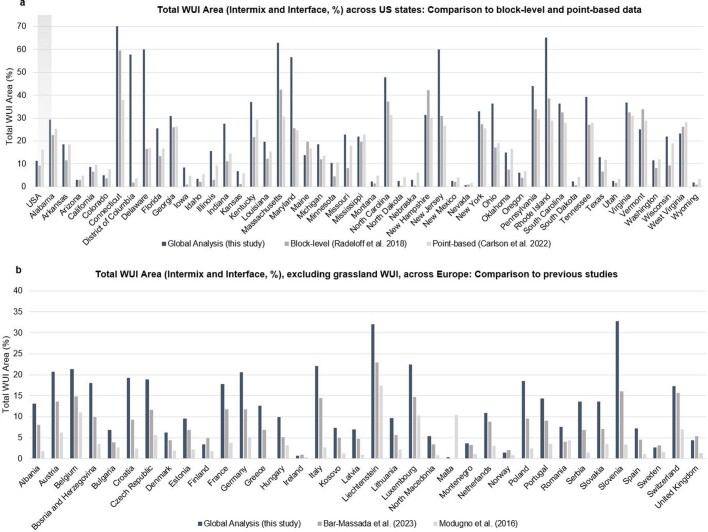
Total WUI area comparison with previous studies states in the conterminous United States and for selected European countries. **a**, Comparison of aggregated global mapping results for 48 conterminous US states to Census block-level and building centroid point-based WUI mapping (Radeloff et al. 2018^19^, Carlson et al. 2022^57^). The data in Radeloff et al. are representative for 2010. The data in Carlson et al. are representative for 2015–2018. **b**, Comparison for 36 European countries: The data in Modugno et al. are representative for 2006^20^. The data in Bar-Massada et al. are representative for 2019–2020^72^. Neither European study considers grassland areas as wildland vegetation. Differences in WUI area share are due to smaller distance thresholds for WUI Interface mapping (600 m distance to closest large vegetation patch in Bar-Massada et al.^73^) and lower mapping resolution with a simplified buffering approach for building detection (only buffer overlaps of built-up land cover and vegetation are considered WUI in Modugno et al.^20^).

We also compared our global WUI map with previously generated census-based and building location-based WUI maps across the United States^19,54^ and found high agreement in total WUI area (Pearson correlation of 0.80) (Extended Data Fig. 7a). In densely populated northeastern states (for example, Connecticut, Massachusetts, New Jersey and Rhode Island), we found considerably more WUI area than census-based and building location-based approaches. In most other states, our WUI area estimate is very close to or slightly higher than estimates from the census-based approach and slightly lower than from the building location-based approach. We also compared our map results with data from two previous studies across 36 European countries^20,73^ and found high agreement in total WUI area with ref. ^27^ (*r* = 0.94 across all countries) and medium agreement with ref. ^20^ (*r* = 0.55) (Extended Data Fig. 7b). However, in Europe, we consistently map more WUI than those two studies. Compared to ref. ^73^, we mapped more WUI because our distance threshold for interface mapping is larger (2.4 km versus 0.6 km) and ref. ^20^ defined the WUI as the overlap of the buffers around built-up land cover (200 m buffer) and vegetation (400 m buffer), which resulted in considerably less WUI.

We developed our WUI maps on the basis of a well-established definition of the WUI that was originally developed in the United States and successfully applied in other world regions (for example, Argentina^23,74^ and Poland^21^) However, WUI maps depend on the mapping criteria, especially the radius that is considered when computing mean building density for a given area and the distance to a large vegetation patch that determines the interface WUI. Previous sensitivity analyses confirmed the general suitability of the parameters that we selected, that is, a 500 m radius for density calculations and a 2,400 m distance to a large vegetation patch. In the United States, radii smaller than 500 m make the resulting WUI maps highly sensitive to commission or omission errors in the underlying building dataset, whereas larger radii resulted in minimal changes in WUI area^54^. In Europe, overall WUI area is 25% lower when limiting interface WUI to areas within 600 m of a large vegetation patch compared to 2,400 m but WUI area estimates based on either distance were highly correlated (*R*² = 0.94; ref. ^73^). Because there are no published WUI-mapping thresholds for most parts of the globe, we decided to apply the most established approach across the globe but acknowledge the value of further regionalized research that accounts for local particularities.

The comparison of wildfire area in the WUI and population affected by wildfire between the MODIS Active Fire and the VIIRS Active Fire datasets showed very similar patterns for both. Globally, 3.1% of wildfire area is in the WUI according to MODIS (2013–2020), compared to 3.5% in VIIRS (2013–2020), with a difference of less than 2.6 percentage points in any world region. The slight difference is probably due to the ability of VIIRS to capture smaller fires and potentially more fires in areas located at the MODIS swath edges (see Supplementary Data B–E for more detailed information and comparisons by biome, region, country and subnational administrative units).

## Online content

Any methods, additional references, Nature Portfolio reporting summaries, source data, extended data, supplementary information, acknowledgements, peer review information; details of author contributions and competing interests; and statements of data and code availability are available at 10.1038/s41586-023-06320-0.

### Supplementary information

Supplementary Information This Supplementary Information file contains additional information to further understand processing details that go beyond the methods section.

Supplementary Data This archive contains all results for the accuracy assessment (A), all world regions (B), countries (C), subnational administrative units (D) and biomes (E) in a tabular (.xlsx.) format. It contains shape files of administrative units that can be linked with the tabular data for analysis in a Geographic Information System.

### Source data

Source Data Fig. 2

Source Data Fig. 3

## Figures and Tables

**Extended Data Table 1:**
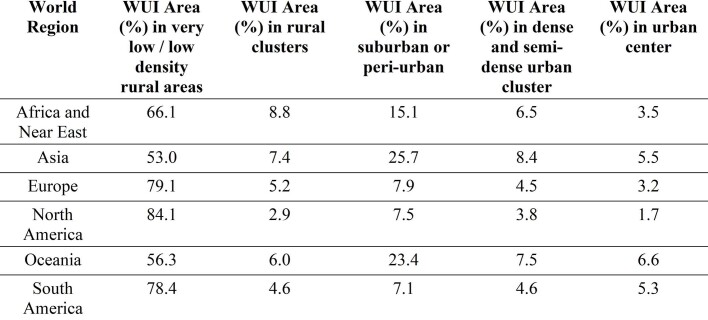
The global Wildland–Urban Interface and the degree of urbanization

WUI area in different degree of urbanization classes. Classes according to Dijkstra et al. (2021)^41^).

**Extended Data Table 2:**
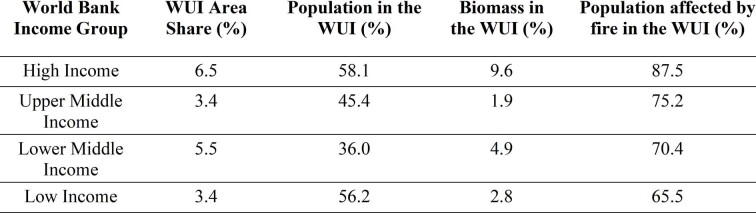
The global Wildland–Urban Interface and income

WUI Area Share, Share of population and biomass in the WUI as well as population affected by fire in the WUI per World Bank Income Group 2023 (https://datahelpdesk.worldbank.org/knowledgebase/articles/906519-world-bank-country-and-lending-groups).

## Data Availability

All raster data are available in a public data repository (https://zenodo.org/record/7941460). The data are also accessible at https://geoserver.silvis.forest.wisc.edu/geodata/globalwui. We share the data for visualization purposes in an interactive data view at https://silvis.forest.wisc.edu/data/globalwui. Source data are provided with this paper.

## Code availability

The algorithm to map the WUI with our raster-based approach is shared here and in the data publication: https://github.com/franzschug/global_wildland_urban_interface.
