# Supplementary material for: The global wildland–urban interface
Source: Nature. 2023 Jul 19;621(7977):94–9. doi: 10.1038/s41586-023-06320-0 (PMC10482693; doi:10.1038/s41586-023-06320-0)
Supplement: Supplementary file 1 — This Supplementary Information file contains additional information to further understand processing details that go beyond the methods section. [file 41586_2023_6320_MOESM1_ESM.pdf]

nature portfolio

https://doi.org/10.1038/s41586-023-06320-0

Supplementary information

The global wildland–urban interface

In the format provided by the authors and unedited

Title: The global wildland-urban interface

Authors: Schug, Franz; Bar-Massada, Avi; Carlson, Amanda R.; Cox, Heather; Hawbaker, Todd J.; Helmers, David; Hostert, Patrick; Kaim, Dominik; Kasraee, Neda K.; Martinuzzi, Sebastián; Mockrin, Miranda H.; Pfoch, Kira A.; Radeloff, Volker C.

Supplementary Information: Datasets and Methods

Land Cover

The *ESA WorldCover* dataset was compiled by the European Space Agency (ESA) as part of the *5th Earth Observation Envelope Programme* (EOEP-5), created by *VITO Remote Sensing*, and is freely and openly provided for download under the Creative Commons Attribution 4.0 International License. Land cover information was derived from using Earth observation data, based on both Copernicus Sentinel-2 spectral-temporal reflectance metrics and temporally aggregated Normalized Difference Vegetation Index (NDVI) data, and Copernicus Sentinel-1 VV and VH radar backscatter time series. The classification was performed using a gradient boosting decision tree algorithm. An ensemble of decision trees was combined with an expert rule based post-processing to derive land cover maps [1].

The land cover data have been independently validated, and have an overall global accuracy of 74.4% [2]. Accuracy for different continents ranges from 67.5% in Oceania to 80.7% in Asia. Globally, 31.5% of all land surface was classified as tree cover, 23.4% as grassland, 17.3% as bare / sparse vegetation, 9.2% as cropland, and 8.6% as shrubland, with user's accuracies (UA) of 80.8% (tree cover), 69.3% (grassland), 87.5% (bare / sparse vegetation), 81.1% (cropland), and 38.6% (shrubland). Urban surfaces were mapped on 0.7% of the global land surface, with a UA of 67.7%. Snow and ice surfaces were mapped with the highest accuracy (93.3% UA, 2.4% area), while herbaceous wetlands were least accurately mapped (27.8% UA, 1.7% area). Beyond the overall good data quality, the authors reported some limitations regarding land cover surfaces potentially relevant for mapping the wildland-urban interface (WUI), for example, a potential confusion of irrigated agriculture and natural wetlands, and between agriculture and highly managed pasture area classified as grassland, as well as a bias towards underestimating impervious area in highly vegetated and sparsely built-up suburban environments. Sentinel-2 orbit/scene overlap and borders or high cloud cover could affect local classification accuracy.

We downloaded the *WorldCover* dataset from the Zenodo data repository [1]. The data were reprojected into the EQUI7 data reference grid and merged into a data cube with all other data using nearest neighbor resampling.

Buildings

The GHS-BUILT-S – R2022A (GHS-BUILT-S) dataset is freely shared by the Joint Research Center of the European Commission under the Creative Commons Attribution 4.0 International License. Here,

building density is approximated by a pixel-wise estimate of built-up area, derived using a symbolic machine learning approach and a 2018 global composite of four Sentinel-2 visible and near-infrared reflectance bands [3]. Symbolic machine learning is a multi-scale approach that uses both reflectance and morphological data, supported by a characteristics-saliency-leveling model [4].

We analyzed the GHS-BUILT-S as a reference for building location and density, acknowledging that the *WorldCover* dataset features a built-up class as well. However, *WorldCover* subsumes buildings and other impervious surfaces, e.g., road infrastructure, in a built-up class, whereas WUI research specifically requires building data, which GHS-BUILT-S provides. Furthermore, as a continuous dataset, GHS-BUILT-S is more sensitive towards detecting small and sparse housing, which is particularly important in WUI mapping, whereas *WorldCover* data are discrete. This discrete character also leads to an underestimation of building density, i.e., an omission of buildings, in suburban and rural areas with low building density, but high vegetation density (e.g., in the form of street trees).

We downloaded the complete GHS-BUILT-S dataset [5], reprojected it into the EQUI7 data reference grid and merged it into a data cube with all other data using bilinear interpolation resampling.

Area Correction

The employed EQUI7 reference grid provides equidistant projections for seven world regions. While minimal, this introduces area distortions across large regions, which impact area statistics. As suggested by and applied in Frantz et al. (2022) [6], we derived a pixel-based area correction factor (up to 15% per world region) to adjust skewed area statistics in order to report true area (Fig. S – 1).

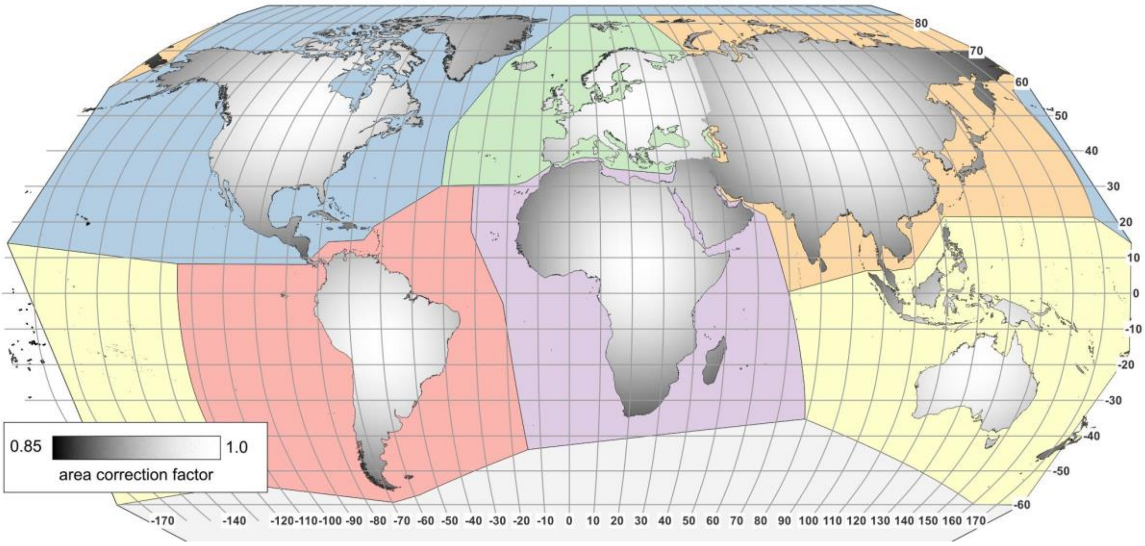

Fig. S – 1 EQUI7 world regions. Pixel-wise area correction factors to adjust skewed area statistics caused by area distortions in the EQUI7 coordinate reference system. Map projection: Robinson. Grid coordinates: WGS 84.

Digital Elevation Model and Slope

We masked steep slopes based on a global digital elevation model composite, based on data captured by the Shuttle Radar Topography Mission (SRTM [7]), and available for download through the U.S. Geological Survey (USGS) EROS Archive [8]. The dataset is provided void-filled (i.e. interpolated data were generated for areas with missing data), with a spatial resolution of 1 x 1 arc-seconds (ca. 30 m at the equator, 1 x 2 arc-seconds north / south of 50° N / S) and a spatial extent from 60° N to 56° S. We filled remaining void areas, particularly north of 60° N, with elevation data from the Advanced Spaceborne Thermal Emission and Reflection Radiometer (ASTER GDEM v3) [9].

Compared to kinematic GPS ground control points, the SRTM elevation model has a mean vertical error of -0.8 to 1.0 m (standard deviation of 5.4 to 9.6 m) globally on land, with a mean geolocation error of < 0.5 m [10]. ASTER GDEM v3 has a mean vertical error of 1.2 m (standard deviation of 8.44 m) compared to U.S. National Geodetic Survey reference GPS data, and an overall bias of -1.81 m compared to the SRTM elevation model [11]. Even though ASTER GDEM v3 improved considerably compared to v2, it still features data artifacts, particularly in extreme latitudes and on ice shields, which have a negligible impact on WUI mapping.

We reprojected elevation data into the EQUI7 data reference grid and merged it into a data cube with all other data using bilinear interpolation resampling. We then derived slope from the digital elevation model with *gdaldem slope* using the *Horn* algorithm [12]. We created a binary slope mask using a 30° threshold, followed by a morphological dilation operation [13] with a 20 m radius, in order to avoid salt and pepper effects or line artifacts along ridges.

Surface Water

We analyzed the Global Surface Water dataset generated by the Joint Research Center under the *Copernicus Programme* [14]. We downloaded global annual *water occurrence* for 2020, representing the duration during which water was present on the surface during a year, via FTP from *jeodpp.jrc.ec.europa.eu***.** The dataset is openly available free-of-charge according to the Copernicus licensing regulations. This dataset is based on Landsat time series data and an expert-based classification system to globally map water at a spatial resolution of 30 x 30 m^2. Water occurrence was computed by dividing the summed water detections by the number of valid observations for each pixel within a month, achieving very high overall accuracies (> 98%) [14]. We reprojected water occurrence data into the EQUI7 data reference grid and merged it into a data cube with all other data using bilinear interpolation resampling.

Wildfire

We used the MODIS Collection 6.1 Terra and Aqua Active Fire Product (MCD14ML; spatial resolution: 1 km) as reference for fire activity across the globe. The active fire dataset detects active fires and other thermal anomalies at the overpass time of the satellite and under relatively cloud-free conditions [15,16]. As part of the Fire Information for Resource Management System (FIRMS), the dataset was initiated by NASA to support global fire monitoring. The active fire product provides continuously detected active fires and thermal anomalies since November 2000 for Terra and since July 2002 for Aqua at 1-2 day intervals. Active fires or other thermal anomalies are categorized into presumed vegetation fires, active volcanos, other static land sources, and offshore detection. The overall global commission error of the product is 1.2% for daytime fire detection, but with regional variability. Fire size is an important limiting factor for fire detection, where small, low-intensity, and rapidly burning fires may be missed, as well as fire in areas with frequent cloud cover [16,17]. As the overall detection of fire is very reliable, we used the dataset as a proxy for fire activity. We downloaded global data from 01st Jan. 2003 to 31st Dec. 2020 from *firms.modaps.eosdis.nasa.gov/download*. We preselected presumed vegetation fires that were captured by both MODIS Terra and Aqua during day and night times. Since the provided confidence values in the dataset should be considered with caution, no further filtering was applied. We also used the VIIRS Active Fire detection data product, also part of the FIRMS program. These data, acquired by the Visible Infrared Imaging Radiometer Suite (VIIRS, launched in 2011) are based on middle and thermal infrared bands and provide active fire points at a nominal spatial resolution of 375 m [18]. We downloaded global data from 01st Jan. 2013 to 31st Dec. 2020 and prepared the data in the same way as MODIS active fire data. We reprojected fire data into the EQUI7 data reference grid.

Processing

We mapped the global extent of the WUI using in-house servers encompassing two machines, both featuring two Intel Xeon E5-2690 with 12 cores / 24 threads and 2.6 GHz CPU speed each, 16 32GB (2133 MT/s) memory modules and running Ubuntu 20.04.4 LTS. We mainly used functionalities of *numpy* [19] and *scipy* [20] for Python 3.10 [21], of the Geospatial Data Abstraction Library (GDAL, v. 3.4), and of the Framework for Operational Radiometric Correction for Environmental monitoring (FORCE [22]). Average building density and land cover area were created using *scipy.ndimage.convolve()* and the *FORCE LandScape Metrics* module. Distance criteria for the categorization of interface WUI were implemented by an identification of large patches (> 5 km^2 of pixels > 75% wildland with and without grassland) using the *FORCE LandScape Metrics* module, followed by a dilation with a 2.4 km buffer size using the *FORCE Texture* module. The different classes of the WUI were derived with a set of computational rules implemented in Python. Please refer to the C*ode Availability* statement for code access.

Validation

Validation sites were randomly selected and stratified based on the mapped area shares of our five classes. An empirically defined minimum distance criterion was applied to avoid multiple sites in spatially autocorrelated areas. We first reclassified the map by aggregating all WUI classes. We then spatially resampled the map to resolution of 1 x 1 km^2 using the mode. For each world region, a random selection of 1,000 EQUI7 tiles was made, from which those with less than 5% mapped overall WUI cover were excluded. We then generated a correlogram computing the global Moran's I, a measure of spatial autocorrelation [23], and according z-values for all selected tiles separately with an increasing lag distance to find a distance threshold where mapping results are no longer spatially autocorrelated. An aggregation of autocorrelation measures suggests that a minimum distance of 30 km between validation sites should avoid spatially autocorrelated samples in most cases (Fig. S - 2).

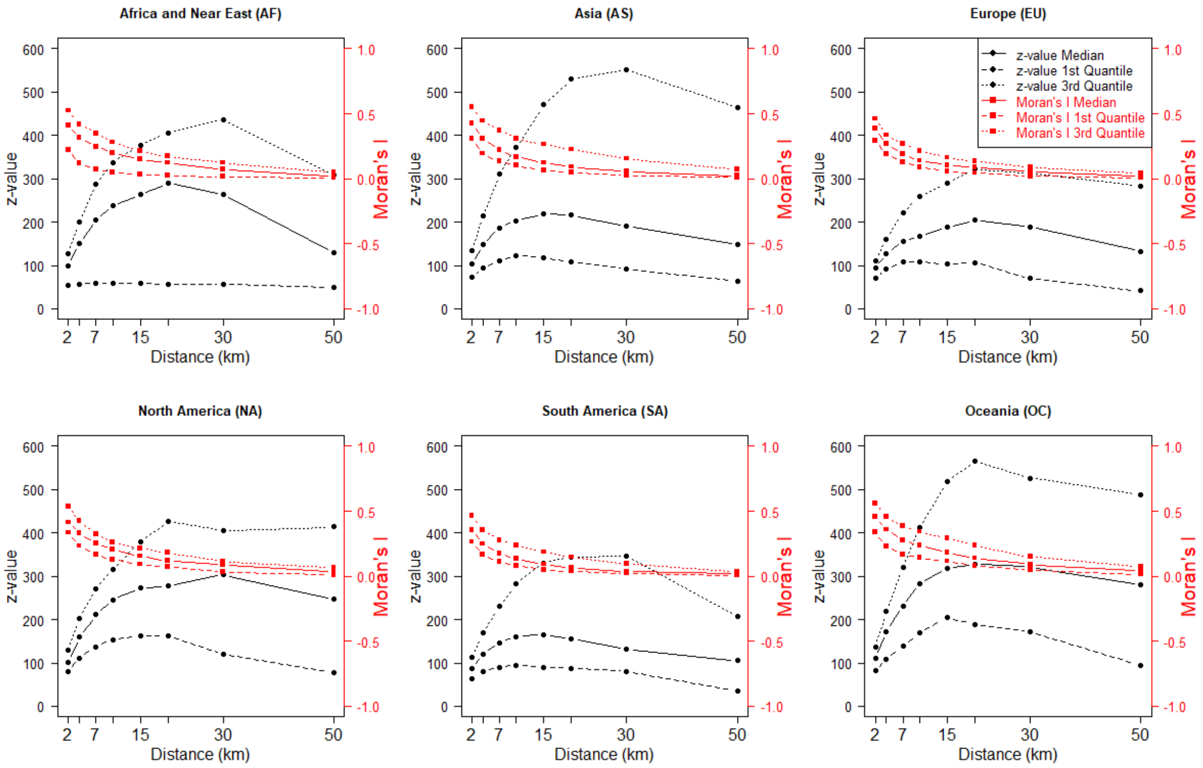

Fig. S - 2 Spatial autocorrelation of the wildland-urban interface maps. Spatial autocorrelation of wildland-urban interface maps for individual world regions. We used the distance where Moran's I and z-value flatten to determine the minimum distance that validation sites need to be apart to avoid spatially autocorrelated validation samples. Sample size: 1,000 tiles (see Extended Data 4 of the manuscript) in each world region.

Reference data for each site were manually generated based on six criteria involving six human expert interpreters and supported by Very High-Resolution imagery accessed via *Google Earth* (https://earth.google.com/web/). While most sites were labelled by a single interpreter each, 60 sites were labelled by all interpreters to assess labeling consistencies among interpreters. Locations with a high labeling uncertainty were tagged correspondingly. For each site, the interpreters decided whether…

… wildland vegetation within a 500 m radius surrounding the site is > 50%

… building area within a 500 m radius surrounding the site is > 0.5%

… building area within a 500 m radius surrounding the site is > 15%

… there is a large wildland patch (≥ 5 km^2, without grassland) within 2.4 km of the site

… there is a large wildland patch (≥ 5 km^2, including grassland) within 2.4 km of the site

Mapping quality was assessed separately for each world region. It was evaluated based on (area-adjusted) overall, user's and producer's accuracy per class.

Class-wise user's and producer's accuracies varied from 75.2% to 87.90% at WUI vs. non-WUI level (see Supplementary Data A). When all five classes (non-WUI, forest/shrub/wetland-dominated intermix WUI, forest/shrub/wetland interface WUI, grassland-dominated intermix WUI, and grassland-dominated interface WUI) were differentiated, class-wise producer's accuracies were between 43.9% (grassland-dominated intermix WUI) and 75.2% (non-WUI) and user's accuracies were between 39.8% (grassland-dominated interface WUI) and 81.9% (non-WUI). We adjusted the accuracy assessment using class-wise mapped area shares in order to reduce the impact of over-represented classes and increase the impact of under-represented classes due to using an equal instead of an area-proportional number of sample sites for all classes in the validation process [24]. The overall accuracy among interpreters differed by similar margins, and a total of 271 points (3%) were tagged uncertain.

There are no definitive reference data for the WUI, and our validation procedure included subjective aspects when interpreting surface conditions that explain the aforementioned differences. For example, the degree of grassland management was difficult to evaluate in many parts of the world but key for whether a surface was classified as wildland or not. Potential interpretation bias could add up to mapping errors in the underlying land cover and building density datasets. As a robustness check, we excluded all validation results from the interpreters that had the best and the worst overall accuracy and found that the final quality estimates did not change considerably.

Disclaimer

Any use of trade, firm, or product names is for descriptive purposes only and does not imply endorsement by the U.S. Government.

References

1. Zanaga, D. *et al.* ESA WorldCover 10 m 2020 v100; 10.5281/zenodo.5571936 (2021).
2. Tsendbazar, N. *et al.* WorldCover Product Validation Report. Available at https://esa-worldcover.s3.amazonaws.com/v100/2020/docs/WorldCover_PVR_V1.1.pdf (2021).

3. Pesaresi, M., Politis, P. GHS built-up surface grid, derived from Sentinel2 and Landsat, multitemporal (1975-2030). European Commission, Joint Research Center (JRC), 10.2905/D07D81B4-7680-4D28-B896-583745C27085, (2022).

4. Pesaresi, M.:, Syrris, V., Julea, A. A New Method for Earth Observation Data Analytics Based on Symbolic Machine Learning. *Remote Sensing,* **8**(5); doi.org/10.3390/rs8050399 (2016).

5. EC. GHSL - Global Human Settlement Layer. Open and free data and tools for assessing the human presence on the planet. Available at https://ghsl.jrc.ec.europa.eu/download.php?ds=buS2 (2022).

6. Frantz, D. *et al.* Material stock map of CONUS; 10.5281/zenodo.6873743 (2022).

7. Farr, T. G. *et al.* The Shuttle Radar Topography Mission. *Reviews of Geophysics* **45**; 10.1029/2005RG000183 (2007).

8. Shuttle Radar Topography Mission (SRTM) 1 Arc-Second Global. Earth Resources Observation and Science Center (2017).

9. Abrams, M., Crippen, R. & Fujisada, H. ASTER Global Digital Elevation Model (GDEM) and ASTER Global Water Body Dataset (ASTWBD). *Remote Sensing* **12,** 1156; 10.3390/rs12071156 (2020).

10. Rodríguez, E., Morris, C. S. & Belz, J. E. A Global Assessment of the SRTM Performance. *Photogrammetric Engineering & Remote Sensing* **72,** 249–260; 10.14358/PERS.72.3.249 (2006).

11. Gesch, D., Oimoen, M., Danielson, J. & Meyer, D. Validation of the ASTER global digital elevation model version 3 over the Conterminous United States. *The International Archives of the Photogrammetry, Remote Sensing and Spatial Information Sciences* **XLI-B4,** 143–148; 10.5194/isprsarchives-XLI-B4-143-2016 (2016).

12. Horn, B. Hill shading and the reflectance map. *Proceedings of the IEEE* **69,** 14–47; 10.1109/PROC.1981.11918 (1981).

13. Soille, P. Erosion and Dilation. In *Morphological Image Analysis,* edited by P. Soille (Springer Berlin Heidelberg, Berlin, Heidelberg, 2004), pp. 63–103.

14. Pekel, J.-F., Cottam, A., Gorelick, N. & Belward, A. S. High-resolution mapping of global surface water and its long-term changes. *Nature* **540,** 418–422; 10.1038/nature20584 (2016).

15. Giglio, L., Descloitres, J., Justice, C. O. & Kaufman, Y. J. An Enhanced Contextual Fire Detection Algorithm for MODIS. *Remote Sensing of Environment* **87,** 273–282; 10.1016/S0034-4257(03)00184-6 (2003).

16. Giglio, L., Schroeder, W. & Justice, C. O. The collection 6 MODIS active fire detection algorithm and fire products. *Remote Sensing of Environment* **178,** 31–41; 10.1016/j.rse.2016.02.054 (2016).

17. Hawbaker, T. J., Radeloff, V. C., Syphard, A. D., Zhu, Z. & Stewart, S. I. Detection rates of the MODIS active fire product in the United States. *Remote Sensing of Environment* **112,** 2656–2664; 10.1016/j.rse.2007.12.008 (2008).

18. Schroeder, W., Oliva, P., Giglio, L., Csiszar, I. A. The New VIIRS 375 m active fire detection data product: Algorithm description and initial assessment. *Remote Sensing of Environment* **143**, 85-96; 10.1016/j.rse.2013.12.008 (2014).

19. Harris, C. R. *et al.* Array programming with NumPy. *Nature* **585,** 357–362; 10.1038/s41586-020-2649-2 (2020).

20. Virtanen, P. *et al.* SciPy 1.0: fundamental algorithms for scientific computing in Python. *Nature methods* **17,** 261–272; 10.1038/s41592-019-0686-2 (2020).

21. van Rossum, G. *The Python language reference.* 3rd ed. (Python Software Foundation; SoHo Books, Hampton, NH, Redwood City, Calif., 2010).

22. Frantz, D. FORCE—Landsat + Sentinel-2 Analysis Ready Data and Beyond. *Remote Sensing* **11,** 1124; 10.3390/rs11091124 (2019).

23. Moran, P. A. P. Notes on Continuous Stochastic Phenomena. *Biometrika* **37,** 17; 10.2307/2332142 (1950).

24. Olofsson, P. *et al.* Good practices for estimating area and assessing accuracy of land change. *Remote Sensing of Environment* **148,** 42–57; 10.1016/j.rse.2014.02.015 (2014).
